# Assessing the druggability of protein-protein interactions by a supervised machine-learning method

**DOI:** 10.1186/1471-2105-10-263

**Published:** 2009-08-25

**Authors:** Nobuyoshi Sugaya, Kazuyoshi Ikeda

**Affiliations:** 1 Drug Discovery Department, Research & Development Division, PharmaDesign, Inc, Hatchobori 2-19-8, Chuo-ku, Tokyo 104-0032, Japan

## Abstract

**Background:**

Protein-protein interactions (PPIs) are challenging but attractive targets of small molecule drugs for therapeutic interventions of human diseases. In this era of rapid accumulation of PPI data, there is great need for a methodology that can efficiently select drug target PPIs by holistically assessing the druggability of PPIs. To address this need, we propose here a novel approach based on a supervised machine-learning method, support vector machine (SVM).

**Results:**

To assess the druggability of the PPIs, 69 attributes were selected to cover a wide range of structural, drug and chemical, and functional information on the PPIs. These attributes were used as feature vectors in the SVM-based method. Thirty PPIs known to be druggable were carefully selected from previous studies; these were used as positive instances. Our approach was applied to 1,295 human PPIs with tertiary structures of their protein complexes already solved. The best SVM model constructed discriminated the already-known target PPIs from others at an accuracy of 81% (sensitivity, 82%; specificity, 79%) in cross-validation. Among the attributes, the two with the greatest discriminative power in the best SVM model were the number of interacting proteins and the number of pathways.

**Conclusion:**

Using the model, we predicted several promising candidates for druggable PPIs, such as SMAD4/SKI. As more PPI data are accumulated in the near future, our method will have increased ability to accelerate the discovery of druggable PPIs.

## Background

Interfering with PPIs by small ligands has been regarded as challenging mainly due to the flatness and large surface area of protein-protein interfaces [1]. However, targeting PPIs is a highly attractive strategy for therapeutic interventions, because most proteins function in cells by interacting with other proteins. To date, over 30 PPIs have been intensively studied as targets for PPI-inhibiting small ligands; these include MDM2/TP53, BCL-X_L_(BCL-2)/BAK, and IL2/IL2 receptor α [[1-7] and references therein]. The interfaces of these drug target PPIs are characterized by a concave, rather than flat, surface and so-called 'hot spots', which is a small area within the interface containing a few amino acids that contribute a large fraction of binding free energy of the interaction [1]. Some PPI-inhibiting small ligands have been proven to have high potency in both *in vitro *and *in vivo *assays on models of human diseases such as cancer [8,9]. These studies strongly support the concept that the PPIs can become therapeutic targets of small molecule drugs.

Since the completion of the human genome sequencing projects, various *in silico *methodologies have been proposed to assess the druggability of all human proteins not yet targeted by drugs and to discover novel drug target proteins. These methods use the 'omics' data of functional, ligand-related, and physicochemical properties of the already-known target proteins [10,11].

In contrast, few methodologies to assess the druggability of PPIs have been proposed. In this era of rapid discovery of PPIs and rapid accumulation of various types of omics data, there is both need and opportunity for development of a methodology that can efficiently select drug target PPIs by holistic assessment of the druggability of PPIs with the omics data. To address this need, we recently proposed an integrative *in silico *approach for discovering druggable PPIs by detecting interacting domains, using Gene Ontology (GO) terms to evaluate similarities in biological function between the two interacting proteins, and finding ligand-binding pockets on the protein surface [12]. Application of our approach to a large body of PPI data showed its effectiveness for assessing the druggability of PPIs and selecting promising candidates for druggable PPIs [12].

To further develop our methodology, we introduced a supervised machine-learning method, SVM, to our integrative approach. Supervised machine-learning methods have been frequently applied to predict the druggability of single drug target proteins [13-17]. In these studies, single proteins targeted by drugs approved by the Food and Drug Administration (FDA) were used as positive instances in the supervised machine-learning. Physicochemical/structural properties [15,16] or functional/ligand-related properties [13,14,17] of these proteins were learned by a machine to produce a learning model suitable for distinguishing single target proteins from other proteins. The model was then applied to all human proteins to predict novel drug targets. These studies have predicted potentially druggable single target proteins with high or moderate accuracies, thus establishing the usefulness of the methods. Here, we apply an SVM-based method to predict novel drug target PPIs. Because the machine-learning-based studies described above strongly suggested the utility of both physicochemical/structural properties and functional/drug-related properties for predicting the druggability of single proteins, we incorporated both types of properties in our SVM methodology.

## Results

**Figure 1:**
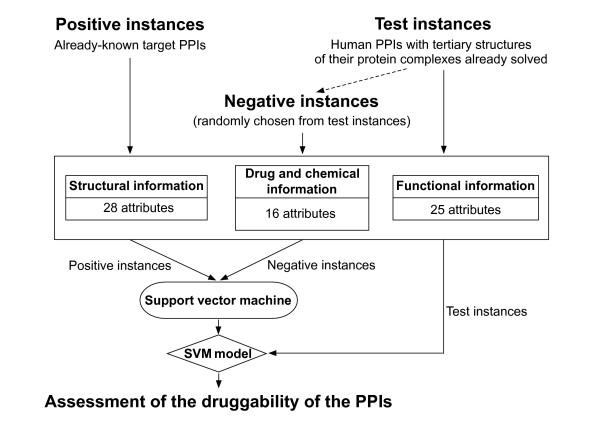
**Schematic representation of the SVM-based method for assessment of the druggability of PPIs**. For the details, see text.

Our approach to assess the druggability of PPIs and predict novel druggable PPIs is schematically represented in Figure 1. To focus on PPIs that may have relevance to human diseases, we assessed only PPIs between human proteins in this study.

### PPI instances

Unlike the previous studies for the prediction of druggable single proteins, our study could not define the PPIs targeted by the FDA-approved drugs as 'druggable' and use them as positive instances in the development of SVM model, because of the scarcity of PPI-inhibiting drugs approved by the FDA. Instead, we adopted PPIs as the positive instances when they satisfy both the following criteria. First, a PPI-inhibiting ligand had been identified and validated by *in vitro *and/or *in vivo *assays that used the two specific partner proteins of the target PPI. Second, a binding pocket for the PPI-inhibiting small ligand was already identified, and it overlaps with the protein-protein interface. The PPI was discarded if small ligands were reported to inhibit the PPI via allosteric effects. Such PPIs were carefully selected from review articles focusing on druggable PPIs [1-7].

Thirty PPIs were selected satisfying the criteria described above (Table 1 and Additional file 1: Table S1). Twelve of these PPIs have tertiary structures of protein/ligand complexes in the PDB database [18]; we used the structures to detect ligand-binding pockets. The remaining PPIs did not have tertiary structures of protein/ligand complexes solved, but their model structures were virtually constructed in the original papers. For these PPIs, we used the structures of protein/protein complexes to detect ligand-binding pockets (see Methods). Forty-one PDB entries (67 polypeptide chains and 5 chain pairs) were used to detect ligand-binding pockets. For any PPI that had more than one similar tertiary structure, all of the structures were considered. As a consequence, 98 instances were used as the positive instances (Additional file 2).

**Table 1:** Positive set PPIs for the SVM-based method. ^a^

PPI		PPI	
	
Protein 1	Protein 2	Protein 1	Protein 2
ARF1	CYTH1 ^b^	GRB2	EGFR
ARF1	CYTH1 ^b^	GRB2	MET
ARF1	CYTH2 ^c^	HOXB1	PBX1
ARF1	CYTH2 ^c^	IL1B	IL1R1
BCL2	BAK1	IL2	IL2RA
BCL2L1	BAK1	MAGI3	PTEN
BIRC4	CASP3	MDM2	TP53
BIRC4	CASP9	PIK3R1	PDGFRB
BIRC4	DIABLO	RAC1	TIAM1
BIRC5	BIRC5	RAC1	TRIO
CALM1	CAMK1	STAT3	STAT3
CALM1	MYLK	TCF7L1	CTNNB1
CALM1	PDE1A	TCF7L2	CTNNB1
CD4	HLA-DQB1	THRB	NCOA2
ESR1	NCOA2	TNF	TNF
FKBP1A	TGFBR1	ZAP70	CD247

^a^For details, see Additional file 1: Table S1.

^b, c^The ligand-binding pockets considered are different from each other.

To obtain test set for the SVM-based method, we retrieved 28,077 human PPIs from the Entrez Gene database [19]. Because our method takes physicochemical/structural properties of protein/protein complexes into consideration, we limited the test set to human PPIs with tertiary structures of their protein complexes already solved. Among the 28,077 PPIs, 1,324 had their tertiary structures, or tertiary structures of similar protein/protein complexes, in the PDB database. Twenty-nine PPIs in the positives were removed (one of the 30 positive set PPIs had no tertiary structure of the protein/protein complex in PDB), and the remaining 1,295 PPIs were considered in this study. These 1,295 PPIs had 6,656 non-redundant PDB entries (8,750 polypeptide chain pairs) showing amino acid sequence similarity of ≥80% to the protein complexes. For any PPI that had more than one similar tertiary structure, all of the structures were considered. As a consequence, we used 10,915 instances as the test instances in the following studies (Additional file 3). In addition, randomly chosen subsets of the test instances were used as negative instances in training data.

### Selection of the best SVM model

For each instance, we calculated 69 attributes encompassing structural, drug and chemical, and functional information (Table 2; see Additional file 4: Supplementary Methods for the definitions and calculation methods for the attributes) and used them as feature vectors in the SVM-based method. Attributes of all instances calculated can be obtained from Additional files 2 and 3.

**Table 2:** Attributes of the PPIs used in the SVM-based method. ^a^

No.	Attribute
	Structural information
1	Pocket volume
2	Accessible surface area of pocket
3	Percentage of accessible surface area of pocket to that of total surface of protein
4	Pocket compactness
5	Pocket planarity
6	*d*_*1*_+*d*_*2*_
7	Pocket narrowness
8	*d*_*4*_+*d*_*5*_
9	Ratio of Ala frequency on pocket surface to that on total surface ^b^
10	Ratio of Cys frequency on pocket surface to that on total surface ^b^
11	Ratio of Asp frequency on pocket surface to that on total surface ^b^
12	Ratio of Glu frequency on pocket surface to that on total surface ^b^
13	Ratio of Phe frequency on pocket surface to that on total surface ^b^
14	Ratio of Gly frequency on pocket surface to that on total surface ^b^
15	Ratio of His frequency on pocket surface to that on total surface ^b^
16	Ratio of Ile frequency on pocket surface to that on total surface ^b^
17	Ratio of Lys frequency on pocket surface to that on total surface ^b^
18	Ratio of Leu frequency on pocket surface to that on total surface ^b^
19	Ratio of Met frequency on pocket surface to that on total surface ^b^
20	Ratio of Asn frequency on pocket surface to that on total surface ^b^
21	Ratio of Pro frequency on pocket surface to that on total surface ^b^
22	Ratio of Gln frequency on pocket surface to that on total surface ^b^
23	Ratio of Arg frequency on pocket surface to that on total surface ^b^
24	Ratio of Ser frequency on pocket surface to that on total surface ^b^
25	Ratio of Thr frequency on pocket surface to that on total surface ^b^
26	Ratio of Val frequency on pocket surface to that on total surface ^b^
27	Ratio of Trp frequency on pocket surface to that on total surface ^b^
28	Ratio of Tyr frequency on pocket surface to that on total surface ^b^
	
	Drug and chemical information
29	Number of small chemical drugs (*L*) ^d^
30	Number of small chemical drugs (*S*) ^e^
31	Number of biotech drugs (*L*) ^d^
32	Number of biotech drugs (*S*) ^e^
33	Number of approved drugs (*L*) ^d^
34	Number of approved drugs (*S*) ^e^
35	Number of experimental drugs (*L*) ^d^
36	Number of experimental drugs (*S*) ^e^
37	Number of investigational drugs (*L*) ^d^
38	Number of investigational drugs (*S*) ^e^
39	Number of nutraceutical drugs (*L*) ^d^
40	Number of nutraceutical drugs (*S*) ^e^
41	Number of withdrawn drugs (*L*) ^d^
42	Number of withdrawn drugs (*S*) ^e^
43	Number of illicit drugs (*L*) ^d^
44	Number of illicit drugs (*S*) ^e^
	
	Functional information
45	Both proteins are related to OMIM-registered diseases (1) or not (0)
46	Number of interacting proteins (*L*) ^d^
47	Number of interacting proteins (*S*) ^e^
48	Number of biological pathways in which either protein is involved (*L*) ^d^
49	Number of biological pathways in which either protein is involved (*S*) ^e^
50	Number of biological pathways in which both interacting proteins are involved
51	Identity scores of the GO terms in the Cellular Component category
52	Identity scores of the GO terms in the Molecular Function category
53	Identity scores of the GO terms in the Biological Process category
54	Number of paralogs in the KEGG (*L*) ^d^
55	Number of paralogs in the KEGG (*S*) ^e^
56	Number of paralogs in the PIRSF (*L*) ^d^
57	Number of paralogs in the PIRSF (*S*) ^e^
58	Number of gene-expressing health states (*L*) ^d^
59	Number of gene-expressing health states (*S*) ^e^
60	Number of health states in which both genes are expressed
61	Number of gene-expressing body sites (*L*) ^d^
62	Number of gene-expressing body sites (*S*) ^e^
63	Number of body sites in which both genes are expressed
64	Number of gene-expressing developmental stages (*L*) ^d^
65	Number of gene-expressing developmental stages (*S*) ^e^
66	Number of developmental stages in which both genes are expressed
67	Similarity scores of gene expression profiles in the Health State category
68	Similarity scores of gene expression profiles in the Body Sites category
69	Similarity scores of gene expression profiles in the Developmental Stage category

^a^For details of the definitions and calculation methods, see Additional file 4: Supplementary Methods.

^b^Abbreviations: Ala, alanine; Cys, cysteine; Asp, aspartic acid; Glu, glutamic acid; Phe, phenylalanine; Gly, glycine; His, histidine; Ile, isoleucine; Lys, lysine; Leu, leucine; Met, methionine; Asn, asparagine; Pro, proline; Gln, glutamine; Arg, arginine; Ser, serine; Thr, threonine; Val, valine; Trp, tryptophan; Tyr, tyrosine.

^d^Defined as the larger one of the two numbers for the two interacting proteins in a PPI.

^e^Defined as the smaller one of the two numbers for the two interacting proteins in a PPI.

We first developed multiple SVM models and used cross-validation to test which model was most suitable for assessing the druggability of PPIs. The cross-validation tests were conducted with the four kernel functions (linear, polynomial, radial basis function (RBF), and sigmoid) for three types of training data (ratios of positive instances:negative instances 1:1, 1:2, and 1:3). We created 10,000 random training data sets (composed of randomly chosen positives and randomly chosen negatives) and calculated average values of accuracy, sensitivity, and specificity (see Methods).

**Figure 2:**
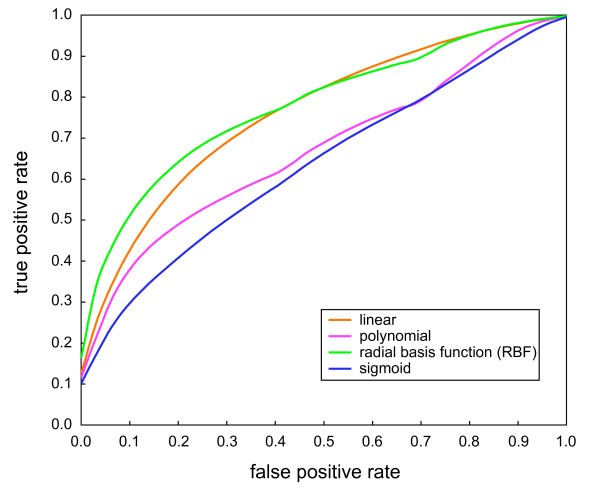
**ROC curves of the training data with the SVM model using all 69 PPI attributes (1:1 positives:negatives ratio)**. ROC curves with the linear (orange), polynomial (magenta), RBF (green), and sigmoid (blue) kernels were calculated for the 10,000 random training data sets, and average values of true positive rate at each false positive rate are plotted. AUCs ± standard deviations of the ROC curves with the linear, polynomial, RBF, and sigmoid kernels are 0.76 ± 0.09, 0.67 ± 0.20, 0.78 ± 0.13, and 0.64 ± 0.17, respectively.

With all three types of the training data, the highest accuracies and highest specificities were obtained from SVM models with the RBF kernel (Table 3). The models with the linear or polynomial kernel followed those with the RBF kernel, showing similar but slightly lower accuracies, and those with the sigmoid kernel had the lowest. When sensitivities were compared, the highest values with the training data ratio of 1:1 (positives:negatives) were obtained by the SVM models with the RBF kernel, and the highest sensitivities with ratios of 1:2 and 1:3 were obtained by the models with the linear kernel. Therefore, on the whole, the models with the RBF kernel seemed to be more suitable for discriminating between the positive and negative instances used here. Receiver operating characteristic (ROC) curves by the SVM models supported this result. Figure 2 clearly shows that area under the curve (AUC) for the ROC curves was the largest for the model with the RBF kernel followed by the polynomial and linear kernels.

**Table 3:** Summary of the results of the cross-validation tests.

Kernel function		Positives:negatives			
		
		All attributes			Top 10 attributes by F-score
		
		1:1	1:2	1:3	1:1
Linear	Accuracy	72.05 ± 6.40	75.37 ± 4.75	79.22 ± 3.78	74.91 ± 5.96
	Sensitivity	71.54 ± 8.97	65.73 ± 7.80	60.21 ± 8.48	75.34 ± 8.19
	Specificity	72.56 ± 8.31	80.19 ± 4.96	85.56 ± 3.96	74.47 ± 8.14
					
Polynomial	Accuracy	70.86 ± 8.83	76.18 ± 6.06	81.18 ± 3.98	71.74 ± 7.73
	Sensitivity	79.85 ± 9.15	53.35 ± 28.74	52.38 ± 25.58	83.29 ± 10.58
	Specificity	61.87 ± 18.47	87.60 ± 8.01	90.78 ± 5.49	60.19 ± 20.23
					
Radial basis function	Accuracy	80.50 ± 4.33	83.43 ± 3.22	86.37 ± 2.36	81.53 ± 4.36
	Sensitivity	81.61 ± 5.84	65.18 ± 9.37	58.67 ± 10.09	82.76 ± 6.09
	Specificity	79.40 ± 6.64	92.55 ± 3.61	95.61 ± 2.46	80.29 ± 6.51
					
Sigmoid	Accuracy	63.79 ± 10.87	69.68 ± 7.73	73.30 ± 6.97	63.32 ± 14.62
	Sensitivity	62.62 ± 16.32	31.69 ± 23.08	23.51 ± 19.63	61.37 ± 18.06
	Specificity	64.96 ± 16.95	88.67 ± 10.62	89.90 ± 8.93	65.27 ± 17.23

Numbers shown are average percentage ± standard deviation.

Table 3 also shows that the more negative instances were included in the training data, the higher the accuracies obtained were in the cross-validation. However, sensitivities and specificities were unbalanced in the training data of 1:2 and 1:3 positives:negatives ratio. While specificities gradually increased in all kernels with the inclusion of more negative instances, sensitivities drastically decreased. This indicates that when a SVM model is trained with more negative instances in the training data, the model has higher power to correctly judge a novel negative instance to be negative, but the power to judge a novel positive instance to be positive rapidly decreases. Sensitivities and specificities of the SVM model with the RBF kernel using the training data of 1:1 positives:negatives ratio were balanced with each other, suggesting that this model had the best combined discriminative power for both the positive and negative instances. Thus, we adopted it as the SVM model most suitable for the assessment of the druggability of the PPIs and used it in the following studies.

### Discriminative power of the attributes

**Figure 3:**
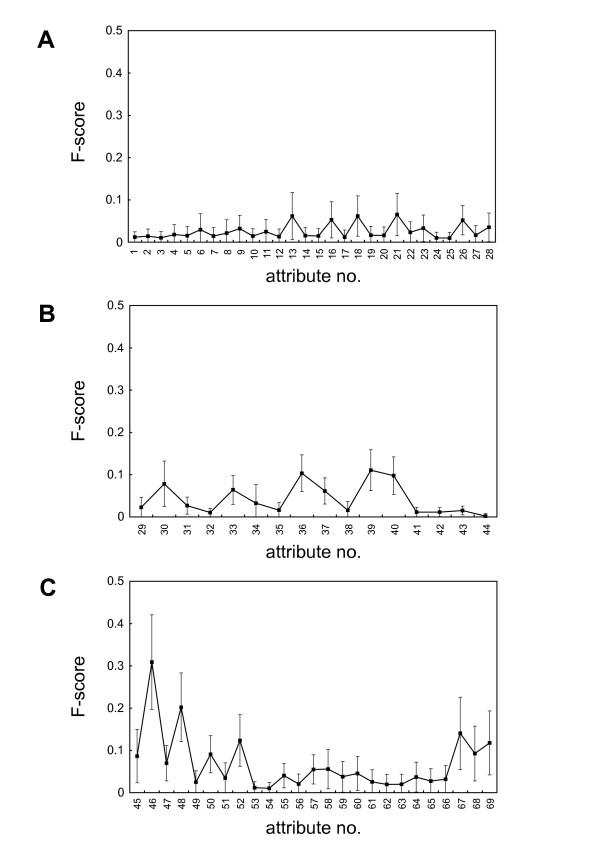
**F-scores of the (A) structural, (B) drug and chemical, and (C) functional attributes**. Average values (black squares) and standard deviations (vertical lines passing through the squares) are shown. For descriptions of the attributes, see Table 2.

To what degree does each attribute contribute to the discrimination between the positive and negative instances in the best model? To evaluate discriminative power of each attribute, we calculated the feature score (F-score) [16,20] of each PPI attribute. A larger F-score indicates that the attribute is more likely to be discriminative [16]. F-scores were calculated for all 10,000 random training data sets. Averages and standard deviations for the attributes were plotted in Figure 3.

Interestingly, the degrees of discriminative power considerably differed among the three types of attributes. In general, although the structural and the drug and chemical attributes had low to medium F-scores (Figures 3A and 3B), the functional attributes had higher F-scores (Figure 3C). Seven of the top 10 highest F-scores (in descending order, attribute no. 46, 48, 67, 52, 69, 39, 36, 40, 68, and 50) were functional attributes (Additional file 1: Table S2). This implies that information on biological function could be the most discriminative for selecting PPIs as drug targets.

**Figure 4:**
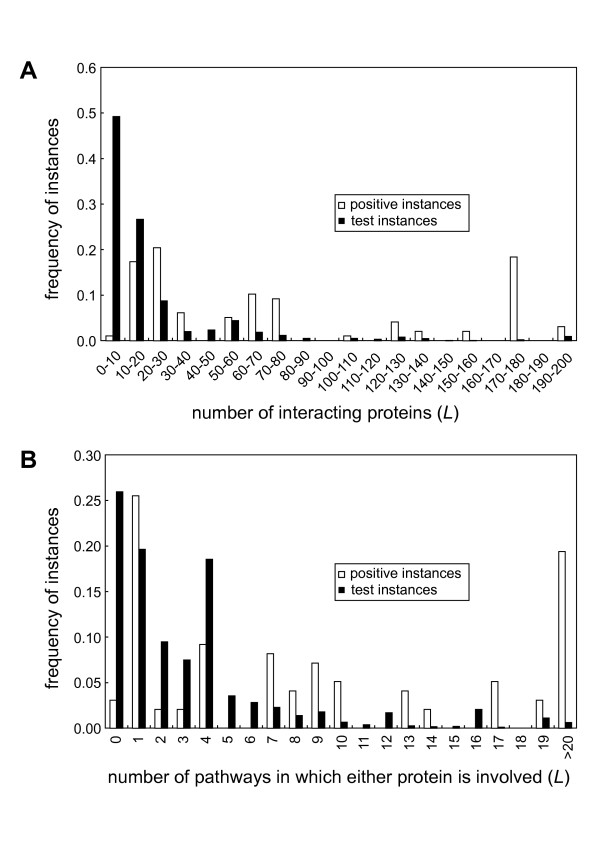
**Frequency distributions of (A) the number of interacting proteins (*L*) (attribute no. 46) and (B) the number of pathways in which either protein is involved (*L*) (attribute no. 48)**. For both attributes, the difference between the frequency distributions of the positive and test instances is statistically significant (*P *< 10^-15^) by the two-sample Kolmogorov-Smirnov test.

Another remarkable point is that, among all attributes, the most discriminative was attribute no. 46 (number of interacting proteins (*L*)), followed by no. 48 (number of pathways in which either protein is involved (*L*)), both functional attributes (Figure 3C, Table S2). Frequency distributions of these two attributes indicate that, on average, the already-known target PPIs had at least one of the two proteins with larger numbers of interacting proteins and with larger numbers of pathways in which the protein is involved than did other PPIs (Figure 4). PPIs, for which at least one partner protein interacts with many additional proteins in the PPI network and exerts versatile functions in multiple pathways, may be suitable as drug targets (see Discussion).

The structural attributes with the highest F-scores were those related to frequencies of certain amino acids (phenylalanine (no. 13), isoleucine (no. 16), leucine (no. 18), valine (no. 26), and proline (no. 21)) (Figure 3A). The drug and chemical attributes with the highest F-scores were no. 36 (number of experimental drugs (*S*)), 39 (number of nutraceutical drugs (*L*)), and 40 (number of nutraceutical drugs (*S*)).

To evaluate whether selection of the attributes according to their F-scores can influence the discriminative power of the best SVM model, we performed cross-validation using the 10 attributes with the highest F-scores (Table 3). The training data with 1:1 positives:negatives ratio was used for the test. Table 3 shows that accuracies, sensitivities, and specificities were all nearly the same as those from using all attributes with 1:1 positives:negatives ratio. This indicates that limiting the attributes to the top 10 by F-score had a limited influence on the discrimination between the positives and negatives, and the SVM model based on the top 10 attributes had a discriminative power not inferior to that based on all attributes. Therefore, we determined to use two types of attribute combinations, all 69 attributes and the top 10 attributes by F-score, to assess the druggability and predict novel druggable PPIs in the next section.

### Prediction of novel druggable PPIs

To predict novel druggable PPIs, the SVM models trained by each of the 10,000 random training data sets were applied to the positive and test instances. We counted the number of times an instance was judged to be positive in the 10,000 training-prediction reiterations. This number is called 'druggability score' hereafter.

**Figure 5:**
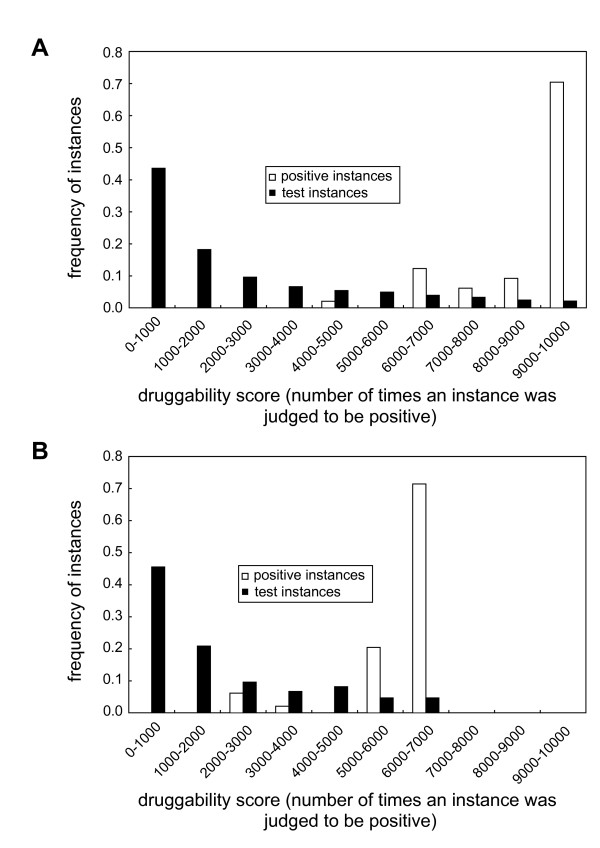
**Frequency distributions of the druggability scores (the number of times an instance was judged to be positive) by the SVM models using (A) all attributes and (B) the top 10 attributes by F-score**.

Frequency distributions of the druggability scores by the SVM models indicate that the positive and test instances were well separated by the models (Figure 5). Among the 10,915 test instances, 69 instances (42 PPIs) had the druggability scores of >9,000 by the SVM models using all attributes and >6,500 by the SVM models using the top 10 attributes (Table 4 and Additional file 1: Table S3). The thresholds of 9,000 and 6,500 were arbitrarily set based on averages of the frequency distributions of the positive instances. The complete prediction results are shown in Additional file 5.

**Table 4:** Potentially-druggable PPIs predicted by the SVM-based method. ^a^

PPI		PPI	
	
Protein 1	Protein 2	Protein 1	Protein 2
APC	CTNNB1	CTNNB1	CTNNBIP1
ARHGAP1	CDC42	E2F2	RB1
ARHGDIA	CDC42	EGFR	ERRFI1
ARHGDIA	RAC1	EP300	CITED2
ARHGDIA	RAC2	EP300	HIF1A
BCL2L1	BECN1	EP300	MYB
BCL9	CTNNB1	GSK3B	AXIN1
CALM1	KCNN2	HRAS	RALGDS
CALM1	RYR1	HRAS	RASA1
CALM2	MARCKS	MAX	MYC
CD247	SHC1	NCF2	RAC1
CDC42	ITSN1	NFKB1	TXN
CDC42	MCF2L	NFKBIB	RELA
CDC42	WAS	RAC1	ARFIP2
CDH1	CTNNB1	RAF1	RAP1A
CREBBP	CITED2	RPA1	TP53
CREBBP	HIF1A	S100B	TP53
CREBBP	IRF3	SMAD2	ZFYVE9
CREBBP	MYB	SMAD4	SKI
CTNNA1	JUP	TP53	TP53BP1
CTNNB1	BTRC	TP53	TP53BP2

^a^For details, see Additional file 1: Table S3. PPIs were listed if an instance of the PPIs had the druggability scores of >9,000 by the SVM model using all attributes and >6,500 by the model using the top 10 attributes by F-score.

The PPIs predicted to be potentially druggable are highly varied with respect to their biological function and cellular location: membrane receptor/signaling adapter (CD247/SHC1) and calmodulin/ion channel (CALM1/KCNN2 and CALM1/RYR1) located on membrane, GTPases/their regulators (CDC42/ARHGDIA, HRAS/RALGDS, *etc*) and kinase/its regulator (GSK3B/AXIN1) in cytoplasm, and histone acetyltransferases/transcriptional regulators (CREBBP/HIF1A, CREBBP/IRF3, EP300/HIF1A, *etc*) and transcription factors/their regulators (MAX/MYC, S100B/TP53, TP53/TP53BP1, *etc*) in the nucleus (Table 4 and Additional file 1: Table S3). Among the PPIs listed, in approximately, neither interacting protein was included in the positives, while in the remaining half, one of the interacting proteins was included in the positives. Therefore, the SVM models used here are not over-trained by the positives and have a predictive power adequate to discover novel druggable PPIs. Interestingly, for some of the PPIs predicted to be druggable, such as MYC/MAX and EP300/HIF1A, small ligands inhibiting the PPIs have been already discovered [3,6], but they were not included in the positives because tertiary structures of the protein/ligand complexes had not yet been solved. This result strongly suggests that our approach is very effective in predicting druggable PPIs.

## Discussion

### Comparison with other studies

In this study, we applied a supervised machine-learning method, SVM, to assess the druggability of human PPIs. Because of lack of information on what attributes are most responsible for PPI druggability, our approach adopted PPI attributes representing various types of information on the structures of the PPIs and their constituent proteins, drugs/chemicals related to the PPIs, and biological function. The best SVM model constructed here can distinguish the already-known target PPIs (positive instances) from others (test instances) with an accuracy of 81%. This value is comparable to the accuracies (75–85%) obtained in the previous studies on the druggabilities of single proteins [13-17]. The accuracies obtained here imply that the set of attributes adopted have a discriminative and predictive power not inferior to the attribute sets used in the previous studies.

In our previous study, we have proposed an integrative method for discovering druggable PPIs by using only the three attributes; the presence/absence of interacting domains, the identity scores of GO terms, and the presence/absence of ligand-binding pocket(s) on the surface of either of the two interacting proteins (not limited to PPI interface) [12]. We applied this method to our original PPI data obtained from the high-throughput yeast two-hybrid experiments [12]. The SVM-based method proposed here was not applied to the PPI data set used in our previous study, because most of these PPIs had no tertiary structure of protein/protein complex satisfying the threshold of sequence similarities of =80%. Several PPIs, similar to the 6 PPIs (RXRA/NRIP1, PPARA/RXRA, RXRB/PPARD, STAT1/STAT6, CDK2/CDKN1A, and STAT3/DST) considered as potentially-druggable in [12], were included in the PPI data set used here. Some PPIs showed moderate or high druggability scores. For example, RXRA/NCOA1, RXRA/PPARBP, and RXRA/NCOA2 similar to RXRA/NRIP1 had the druggability scores of 4,618~9,155 by the SVM model using all attributes (Additional file 5). The druggability scores of PPARG/RXRA, similar to PPARA/RXRA and RXRB/PPARD, were 4,428~6,227. In contrast, other PPIs showed low druggability scores. These are CDK2/CDKN1B (2,769; similar to CDK2/CDKN1A) and STAT1/STAT1 (839; similar to STAT1/STAT6). Among the attributes used in [12], only the identity score of GO terms in the Molecular Function category has high F-score, and the numbers of interacting proteins and pathways showing highest F-scores were not used in the previous study. The disagreement between the results in [12] and in the present study may be ascribed to the PPI attributes not used in [12]. To realize consistent assessment of the druggability, it will be needed to discover an attribute or a combination of attributes that are most ideal for the assessment.

### Contribution of the attributes to the discrimination

Based on the F-scores calculated, the most discriminative attributes by which a PPI is judged to be positive are the number of interacting proteins and the number of pathways (for the larger one of the two numbers for the two interacting proteins in a PPI). This is partially in agreement with the results in [17,21,22], which showed that drug target proteins are 'more highly connected in PPI networks and biological pathways (but far from being the most highly connected)'. One possible explanation is that these characteristics are simply a result of the intensive study that proteins or PPIs have received in the course of development of drugs targeting them. Intensively studied proteins and PPIs may have more interacting proteins and be involved in more pathways discovered than do proteins and PPIs that have received less study. However, we favor the hypothesis that large numbers of interacting proteins and pathways are characteristics intrinsic to good drug targets. From studies on the topology of scale-free networks, including PPI networks and biological pathways, it is well known that disrupting highly-connected nodes is most detrimental to those networks [23,24]. Highly-connected proteins therefore make good targets for the purpose of repressing biological pathways related to disease. Thus, the PPIs selected as targets tend to be those that have versatile biological functions via additional interactions with various other proteins and participation in various pathways in the cell.

Other top 10 attributes by F-score include the similarity scores of gene expression profiles, the identity score of GO terms, and the numbers of drugs in some drug categories (Table S2). Frequency distributions of these attributes show that the positive instances tend to have lower similarity scores of gene expression profiles in the three UniGene categories, lower identity scores of GO terms in the Molecular Function category, and the smaller numbers of nutraceutical and experimental drugs (data not shown). Lower similarity scores and identity scores of the positive instances should be due to higher frequency of heterodimers in the positives. As shown in Table 1, most of the already-known target PPIs are heterodimers. In contrast, the test instances contain the large number of homodimers (53.9%; 5,880/10,915 instances). It is natural that homodimers tend to have higher similarity scores of gene expression profiles and higher identity scores of GO terms than do heterodimers, because gene expression profiles and GO terms are perfectly identical between the same proteins. This result implies that heterodimers have been more preferred as drug targets. As for the smaller numbers of nutraceutical and experimental drugs, this might simply reflect the fact that the interacting proteins of the already-known target PPIs have not been targeted for these drugs so far.

Unexpectedly, the contribution of most structural attributes to the discrimination between the positive and negative instances is smaller than that of the functional attributes and drug and chemical ones. There is no structural attribute in the top 10 by F-score. Because tertiary structures of target proteins are essential to computationally design small molecule drugs based on the structures, this result seems to be not compatible with intuition of researchers studying *in silico *drug design. The contribution of structural information may be hidden by the large contribution of the functional attributes such as the numbers of interacting proteins and pathways. Among the structural attributes, however, those related to frequencies of hydrophobic amino acids (phenylalanine, isoleucine, leucine, and valine) show relatively higher F-scores. Frequency distributions of the attributes indicate that the frequency ratios of these hydrophobic amino acids in the surface of the ligand-binding pockets tend to be higher in the already-known target PPIs than in the non-target PPIs (data not shown). This implies that, when the two groups of the pockets are compared with each other, hydrophobic amino acids more preferentially occur on the surfaces of the former pockets than the latter. This is in good agreement with the results in previous studies [25,26] suggesting that druggable pockets are generally composed of hydrophobic amino acids. It is likely that the interfaces composed of more hydrophobic amino acids than other types of amino acids are particularly amenable targets for PPI-inhibiting small chemicals.

### Comparison between the two SVM models

To predict novel druggable PPIs, we used two types of attribute combinations, all 69 attributes and the top 10 attributes by F-score, having nearly the same degree of discriminative power with respect to accuracies, sensitivities, and specificities. This seems to be redundant on the surface, but the SVM models using the two combination types yield prediction results different from each other. As shown in Figure 5, the SVM model using all attributes separates the positive instances from the test instances better than the model using the top 10 attributes does. In addition, there is low correlation (correlation coefficient 0.57) between the druggability scores by the SVM model using all attributes and the scores by the model using the top 10 attributes. The number of test instances is 163 that have the druggability scores of ≥9,000 by the former model but <6,500 by the latter model. On the one hand, 145 test instances have the druggability scores of <9,000 by the former model but ≥6,500 by the latter model. These results imply that using both models to predict druggable PPIs is necessary not to miss PPIs for which one model predicts to be druggable but other model does not.

### Prospects of our approach

Finally, to validate that our approach is really useful for *in silico *drug design, we performed a pharmacophore analysis to one of the PPIs predicted as potentially-druggable by our method (Table 4). By searching small ligands that have chemical structure similar to the hot spots of the protein-protein interface, one could find candidate ligands that might interfere with the PPI [27]. As an example, we focused on the SMAD4/SKI PPI [PDB:1MR1_BC] (Additional file 6: Figure S3). This PPI had the druggability scores of 9,526 and 6,899 by the SVM models using all attributes and the top 10 attributes, respectively (Additional file 1: Table S3). To our knowledge, no small ligand that inhibits this PPI has yet been reported. In *in silico *drug design, pharmacophore analysis has been frequently used as the first step to search candidates for drug seeds, when no small ligand to a drug target was known. Using a pharmacophore model of the hot spots of the SMAD4/SKI interface (Figure S3D), we searched for small ligands against a subset of drug-like chemicals in the ZINC database [28]. We found 9 small chemicals showing similarities to the hot spots (Additional file 1: Table S4). SKI is an oncoprotein that is frequently overexpressed in some types of cancer such as melanoma [29,30]. It functions as a suppressor of the TGFβ signaling pathway via interference with interactions between SMAD family proteins that act as TGFβ-signal mediators [31]. By suppressing the TGFβ signaling pathway, SKI protein could play an essential role in preventing a cancer cell from differentiating to a defined cell type. If the small chemicals and their derivatives found here have the potential to inhibit SMAD4/SKI interaction by binding to the interface pocket located on SKI, these chemicals may serve as drug seeds for the development of anticancer drugs inhibiting the PPI. As the next step, experimental assays will be needed to validate the potential of these chemicals to inhibit SMAD4/SKI.

Although, in this study, we concentrated on PPIs between human proteins, PPIs of human proteins with parasite, bacterial, or viral proteins and those among the latter proteins may also be crucial drug targets. Indeed, many such PPIs, including Nef/Fyn, FtsZ/ZipA, CD4/HIV-gp120, HPV E1/E2, and CRM1/NES, have been intensively studied as drug targets [1-7]. Because several attributes used here cannot be directly applied to PPIs involving pathogen proteins, we did not study these PPIs. However, introducing attributes suited to PPIs involving pathogen proteins can make our approach fully applicable to these PPIs.

## Conclusion

The size of human interactome has been predicted as approximately 150,000–370,000 PPIs [32]. In contrast, the number of human PPIs in a public database is limited to only about ~40,000 [33]. It is highly probable that PPIs yet to be detected include many druggable PPIs. The approach proposed here will accelerate discovery of the promising candidates for druggable PPIs. A limitation of the approach is that it requires tertiary structures of the protein complexes. The number of the human PPIs with tertiary structures of their protein complexes already solved is only about 1,300. With the accumulation of additional tertiary structures of the protein complexes and the advancement of computer technologies to simulate protein-protein docking, our approach will have increased ability to find novel druggable PPIs.

## Methods

### Positive instances

For the 30 PPIs selected from review articles on druggable PPIs (see Results), we investigated whether tertiary structure of a protein/ligand complex had been already solved or model structure of a protein/ligand complex had been constructed and represented in the original papers. If a tertiary structure of a protein/ligand complex had been already solved, we detected the ligand-binding pocket by the computational program Alpha Site Finder implemented in the software package Molecular Operating Environment [34]. If the tertiary structure of the protein/ligand complex had not been solved but that of the protein/protein complex had been solved, we detected the ligand-binding pocket based on the tertiary structure of the protein/protein complex. Then, we carefully checked by visual inspection whether the pocket detected was located at nearly the same position as described in the protein/ligand model structure in the original paper. The pocket detection was done for all polypeptide chains of the protein/ligand or protein/protein complexes. If any ligand-binding pocket was not found on a polypeptide chain, then the chain was discarded. When one large ligand-binding pocket was detected as two or more small pockets, the two pockets were merged if 50% or more amino acid residues constituting one pocket were shared with those constituting another pocket. When a ligand-binding pocket described in the original paper was identified as two distinct, non-overlapping pockets, both pockets were considered separately as 'pocket 1' and 'pocket 2' (see Additional file 2). Coordinate data of the ligand-binding pockets are shown in Additional file 7. For these pockets, the attributes of structural information were calculated as described in Additional file 4: Supplementary Methods, which also describes the calculation methods of the attributes of the drug and chemical and the functional information.

### Test instances

Human PPI data were downloaded from the Entrez Gene database. To retrieve PPIs with tertiary structures of their protein complexes already solved, we performed sequence-similarity searches of the PDB database using the BLASTP program [35] with the default program parameters except for '-F F' (no mask for low complexity regions). If both the two interacting proteins of a PPI showed amino acid sequence similarities of ≥80% to distinct polypeptide chains in the same PDB entry and the two chains physically contact with each other in the tertiary structure of the protein/protein complex, the PPI and the protein/protein complex was included in the test instances. Protein/protein complexes used are not limited to human proteins when those complexes satisfy the criterion above, because, even if those complexes are not from human, it is highly probable that tertiary structures of those complexes have nearly identical to the tertiary structures of the complexes of human proteins. After all pockets were detected for all polypeptide chains in a PDB entry, only one pocket per every polypeptide chain pairs, having the largest area overlapped with protein-protein interface, was considered in the present study.

### Cross-validation tests

Ten-fold cross-validation tests were performed using the program package Libsvm (version 2.86) [20]. For training data, we created 10,000 random data sets composed of randomly chosen positive instances and randomly chosen negative instances. To avoid over-training of the SVM due to the redundancy of similar instances in the positives, only one instance for each PPI in the positive set was randomly chosen and included as positive instance in the training data. When the randomly chosen positive instance was the only instance representing a PPI (9PPIs (CALM1/PDE1A, CD4/HLA-DQB1, HOXB1/PBX1, IL1B/IL1R1, MAGI3/PTEN, PIK3R1/PDGFRB, RAC1/TRIO, STAT3/STAT3, THRB/NCOA2); see Additional file 2), that instance was always included in the training data. If a positive instance was one of several instances representing a PPI (21PPIs including BCL2/BAK1, ESR1/NCOA2, FKBP1A/TGFBR1, *etc*; see Additional file 2) because of multiple similar tertiary structures, we randomly chose a single instance from those instances to include in the training data. Because we had no knowledge on which PPIs cannot be targeted by small ligands at this time, the negative instances were randomly chosen from the 10,915 test instances. To create positives:negatives ratios of 1:1, 1:2, and 1:3, we included 30, 60, and 90 test instances in each training data.

To construct an SVM model most suitable for predicting druggable PPIs, all four kernel functions (linear, polynomial, RBF, and sigmoid) implemented in the Libsvm package were tested with each of the three positives:negatives ratios in the training data. For every random training data set, the best parameters *C *and γ in the kernel functions were estimated by the parameter selection program in the Libsvm package, and then cross-validation tests were performed. We calculated accuracies, sensitivities, and specificities based on the results of cross-validation. Accuracy was defined as (TP+TN)/(TP+TN+FP+FN), sensitivity as TP/(TP+FN), and specificity as TN/(TN+FP), where TP, TN, FP, and FN are the numbers of true positives, true negatives, false positives, and false negatives, respectively. Averages of sensitivities, specificities, and accuracies of the 10,000 cross-validation were calculated.

## List of abbreviations

AUC: area under the curve; FDA: Food and Drug Administration; GO: Gene Ontology; PPI: protein-protein interaction; RBF: radial basis function; ROC: receiver operating characteristic; SVM: support vector machine.

## Authors' contributions

NS conceived of the study, carried out the analyses based on the SVM-based method, and drafted the manuscript. KI created the pharmacophore model of the hot spots of the SMAD4/SKI complex to search for small chemicals similar to the hot spots.

## Supplementary Material

Additional file 1 **Supplementary tables**. Table S1 lists the positive set PPIs in more details than Table 1 in the text. Table S2 lists the top 10 attributes by F-score. Table S3 lists the potentially-druggable PPIs predicted by the SVM-based method in more details than Table 4 in the text. The 69 instances (42 PPIs) listed have the druggability scores of >9,000 by the SVM models using all attributes and >6,500 by the models using the top 10 attributes. Table S4 lists 9 small chemicals showing similarities to the hot spots of the SMAD4/SKI complex. File format, PDF. Click here for file

Additional file 2 **Positive instances**. The 98 instances are listed with their attributes. Attribute numbers correspond to those in Table 2. Click here for file

Additional file 3 **Test instances**. The 10,915 instances are listed with their attributes. Attribute numbers correspond to those in Table 2. Click here for file

Additional file 4 **Supplementary methods. **Definition and calculation methods of the PPI attributes. Click here for file

Additional file 5 **Assessment of the druggability of the PPIs**. For each positive or test instance, the druggability is assessed by the druggability scores (the numbers of times an instance was judged to be positive more than 9,000 times with the SVM models using all attributes and more than 6,500 times with the SVM models using the top 10 attributes). Click here for file

Additional file 6 **Figure S3**. Discovery of small ligands showing similarities to the hot spots of the SMAD4/SKI complex. Click here for file

Additional file 7 **Ligand-binding pockets of the positive PPIs**. Coordinate data of the ligand-binding pockets of the positive PPIs. Click here for file
