# Supplementary material for: Assessing the druggability of protein-protein interactions by a supervised machine-learning method
Source: BMC Bioinformatics. 2009 Aug 25;10:263. doi: 10.1186/1471-2105-10-263 (PMC2739204; doi:10.1186/1471-2105-10-263)
Supplement: Additional file 1 — Supplementary tables. Table S1 lists the positive set PPIs in more details than Table 1 in the text. Table S2 lists the top 10 attributes by F-score. Table S3 lists the potentially-druggable PPIs predicted by the SVM-based method in more details than Table 4 in the text. The 69 instances (42 PPIs) listed have the druggability scores of >9,000 by the SVM models using all attributes and >6,500 by the models using the top 10 attributes. Table S4 lists 9 small chemicals showing similarities to the hot spots of the SMAD4/SKI complex. File format, PDF. [file 1471-2105-10-263-S1.pdf]

**Table S1: Positive set PPIs for the SVM-based method.**

| Protein 1 | Protein 2 | PDB entries and polypeptide chains used for detecting ligand-binding pockets | | References [f] |
|---|---|---|---|---|
| | | Protein/ligand complex | Model structure | |
| ARF1 | CYTH1 | 1s9d_A, 1s9d_E, 1r8q_A, 1r8q_E, 1r8q_B, 1r8q_F [a] | - | [17] |
| ARF1 | CYTH1 | - | 1r8q_BF, 1s9d_AE, 1r8s_AE [b,c] | [18] |
| ARF1 | CYTH2 | 1s9d_A, 1s9d_E, 1r8q_A, 1r8q_E, 1r8q_B, 1r8q_F [a] | - | [17] |
| ARF1 | CYTH2 | - | 1r8q_BF, 1s9d_AE, 1r8s_AE [b,c] | [18] |
| BCL2 | BAK1 | 2o2f_A, 2o22_A, 2o21_A, 1ysw_A | - | [19,20] |
| BCL2L1 | BAK1 | 2o2n_A, 2o2m_A, 2yxj_A, 2yxj_B, 1ysi_A | - | [19,20] |
| BIRC4 | CASP3 | - | 1i3o_E [e], 1i3o_F [e] | [21] |
| BIRC4 | CASP9 | 1tft_A | 1nw9_A | [22,23] |
| BIRC4 | DIABLO | - | 1g73_C, 1g73_D | [24] |
| BIRC5 | BIRC5 | - | 1e31_A, 1e31_B, 1f3h_A, 1f3h_B, 1xox_A, 1xox_B | [25] |
| CALM1 | CAMK1 | - | 1mux_A [e] | [26,27] |
| CALM1 | MYLK | - | 1mux_A [e] | [26,28] |
| CALM1 | PDE1A | 1qiv_A | - | [28,29] |
| CD4 | HLA-DQB1 | - | 1jl4_D | [30] |
| ESR1 | NCOA2 | - | 3erd_A, 3erd_B | [31] |
| FKBP1A | TGFBR1 | 1fkj_A, 1fkf_A | - | [32-35] |
| GRB2 | EGFR | 1cj1_B, 1cj1_C, 1cj1_D, 1cj1_E, 1cj1_F, 1cj1_G, 1cj1_H, 1cj1_I, 1cj1_J [d] | - | [36,37] |
| GRB2 | MET | 1cj1_B, 1cj1_C, 1cj1_D, 1cj1_E, 1cj1_F, 1cj1_G, 1cj1_H, 1cj1_I, 1cj1_J [d] | - | [36,38] |
| HOXB1 | PBX1 | - | 1b72_B | [39] |
| IL1B | IL1R1 | - | 1itb_B | [40] |
| IL2 | IL2RA | 1py2_A, 1py2_B, 1py2_C, 1py2_D, 1m48_A, 1m48_B, 1m4a_A | - | [41,42] |
| MAGI3 | PTEN | - | 1be9_A | [43] |
| MDM2 | TP53 | 1t4e_A, 1t4e_B, 1ttv_A | - | [44,45] |
| PIK3R1 | PDGFRB | - | 1h9o_A | [46] |
| RAC1 | TIAM1 | - | 1foe_B, 1foe_D, 1foe_F, 1foe_H | [47] |
| RAC1 | TRIO | - | 2nz8_A | [47] |
| STAT3 | STAT3 | - | 1bg1_A | [48] |
| TCF7L1 | CTNNB1 | - | 1g3j_A, 1g3j_C | [49] |
| TCF7L2 | CTNNB1 | - | 1g3j_A, 1g3j_C | [49] |
| THRB | NCOA2 | - | 1bsx_B | [50] |
| TNF | TNF | 2az5_AB, 2az5_CD [c] | - | [51] |
| ZAP70 | CD247 | - | 2oq1_A [e] | [52] |

[a] Brefeldin-binding pocket.

[b] Target pocket in [17]. This pocket is distinct from the brefeldin-binding pocket.

[c] The ligand-binding pockets are composed of atoms derived from both two polypeptide chains.

[d] Polypeptide chains 1cj1_A, 1cj1_K, and 1cj1_L were discarded because the ligand-binding pocket was not detected.

[e] The ligand-binding pockets were detected as two distinct pockets that did not overlap. The two pockets were considered separately.

[f] See Additional file 5: Supplementary references.

**Table S2: Top 10 attributes by F-score.**

| No. | Attribute | F-score |
|---|---|---|
| 46 | Number of interacting proteins (*L*) | 0.309±0.112 |
| 48 | Number of biological pathways in which either protein is involved (*L*) | 0.202±0.081 |
| 67 | Similarity scores of gene expression profiles in the Health State category | 0.140±0.085 |
| 52 | Identity scores of the Gene Ontology terms in the Molecular Function category | 0.123±0.061 |
| 69 | Similarity scores of gene expression profiles in the Developmental Stage category | 0.118±0.076 |
| 39 | Number of nutraceutical drugs (*L*) | 0.110±0.049 |
| 36 | Number of experimental drugs (*S*) | 0.103±0.043 |
| 40 | Number of nutraceutical drugs (*S*) | 0.098±0.045 |
| 68 | Similarity scores of gene expression profiles in the Body Sites category | 0.093±0.065 |
| 50 | Number of biological pathways in which both interacting proteins are involved | 0.091±0.044 |

**Table S3: Potentially-druggable PPIs predicted by the SVM-based method.**

| Protein 1 | Protein 2 | Protein/protein complex | Polypeptide chain in which pocket was detected | Druggability score by the SVM model using all attributes | Druggability score by the SVM model using the top 10 attributes by F-score |
|---|---|---|---|---|---|
| CTNNB1 | BTRC | 1p22_CA | 1p22_A | 9941 | 6918 |
| CALM1 | RYR1 | 2bcx_AB | 2bcx_A | 9940 | 6974 |
| CD247 | SHC1 | 1tce_BA | 1tce_A | 9939 | 6657 |
| CTNNB1 | CTNNBIP1 | 1luj_AB | 1luj_A | 9931 | 6858 |
| EP300 | CITED2 | 1r8u_BA | 1r8u_B | 9919 | 6839 |
| CREBBP | CITED2 | 1r8u_BA | 1r8u_B | 9911 | 6860 |
| RAC1 | ARFIP2 | 1i4d_DB | 1i4d_D | 9885 | 6725 |
| S100B | TP53 | 1dt7_AX | 1dt7_X | 9881 | 6588 |
| S100B | TP53 | 1dt7_BY | 1dt7_Y | 9864 | 6588 |
| BCL9 | CTNNB1 | 2gl7_CA | 2gl7_A | 9859 | 6892 |
| CREBBP | MYB | 2agh_BA | 2agh_B | 9858 | 6849 |
| EGFR | ERRFI1 | 2rfd_BD | 2rfd_D | 9845 | 6799 |
| EGFR | ERRFI1 | 2rfe_BF | 2rfe_B | 9840 | 6799 |
| APC | CTNNB1 | 1v18_BA | 1v18_A | 9833 | 6930 |
| EP300 | MYB | 2agh_BA | 2agh_B | 9832 | 6839 |
| RAC1 | ARFIP2 | 1i4d_DA | 1i4d_D | 9831 | 6725 |
| EP300 | HIF1A | 1l8c_AB | 1l8c_A | 9830 | 6859 |
| APC | CTNNB1 | 1th1_DB | 1th1_B | 9808 | 6930 |
| CREBBP | HIF1A | 1l8c_AB | 1l8c_A | 9796 | 6875 |
| RAC1 | ARFIP2 | 1i4l_DB | 1i4l_D | 9750 | 6725 |
| RAC1 | ARFIP2 | 1i4l_DA | 1i4l_D | 9750 | 6725 |
| E2F2 | RB1 | 1n4m_DB | 1n4m_B | 9721 | 6674 |
| EGFR | ERRFI1 | 2rf9_BD | 2rf9_B | 9721 | 6799 |
| ARHGDIA | RAC1 | 1hh4_EB | 1hh4_E | 9692 | 6934 |
| CDC42 | WAS | 1cee_AB | 1cee_A | 9675 | 6746 |
| CDC42 | ITSN1 | 1ki1_CB | 1ki1_B | 9621 | 6716 |
| E2F2 | RB1 | 1n4m_CA | 1n4m_A | 9616 | 6674 |
| CTNNB1 | CTNNBIP1 | 1m1e_AB | 1m1e_A | 9570 | 6858 |
| CDH1 | CTNNB1 | 1i7w_DC | 1i7w_C | 9542 | 6889 |
| ARHGDIA | RAC1 | 1hh4_DA | 1hh4_D | 9541 | 6934 |
| TP53 | TP53BP2 | 1ycs_AB | 1ycs_B | 9535 | 6645 |
| HRAS | RALGDS | 1lfd_BA | 1lfd_B | 9533 | 6614 |
| EGFR | ERRFI1 | 2rfe_AE | 2rfe_A | 9529 | 6799 |
| SMAD4 | SKI | 1mr1_BC | 1mr1_C | 9526 | 6899 |
| NFKB1 | TXN | 1mdi_BA | 1mdi_A | 9525 | 6867 |
| TP53 | TP53BP1 | 1kzy_AC | 1kzy_C | 9497 | 6603 |

**Table S3:** (continued)

| Protein 1 | Protein 2 | Protein/protein complex | Polypeptide chain in which pocket was detected | Druggability score by the SVM model using all attributes | Druggability score by the SVM model using the top 10 attributes by F-score |
|---|---|---|---|---|---|
| CTNNA1 | JUP | 1dow_AB | 1dow_A | 9494 | 6763 |
| CREBBP | IRF3 | 1zoq_DA | 1zoq_A | 9421 | 6809 |
| CALM1 | KCNN2 | 1qx7_MD | 1qx7_M | 9395 | 6871 |
| NFKBIB | RELA | 1oy3_DC | 1oy3_C | 9363 | 6842 |
| RPA1 | TP53 | 2b3g_AB | 2b3g_B | 9355 | 6554 |
| SMAD2 | ZFYVE9 | 1dev_AB | 1dev_A | 9344 | 6899 |
| CREBBP | MYB | 1sb0_AB | 1sb0_B | 9313 | 6849 |
| EP300 | MYB | 1sb0_AB | 1sb0_B | 9312 | 6839 |
| GSK3B | AXIN1 | 1o9u_AB | 1o9u_A | 9300 | 6901 |
| SMAD2 | ZFYVE9 | 1dev_CD | 1dev_C | 9299 | 6899 |
| CDC42 | MCF2L | 1kzg_BA | 1kzg_B | 9295 | 6704 |
| ARHGAP1 | CDC42 | 1am4_CE | 1am4_C | 9265 | 6716 |
| NCF2 | RAC1 | 1e96_BA | 1e96_A | 9223 | 6957 |
| ARHGAP1 | CDC42 | 2ngr_BA | 2ngr_A | 9220 | 6716 |
| BCL2L1 | BECN1 | 2p1l_AB | 2p1l_A | 9220 | 6837 |
| ARHGAP1 | CDC42 | 1am4_CF | 1am4_F | 9210 | 6716 |
| APC | CTNNB1 | 1th1_CA | 1th1_C | 9190 | 6930 |
| NFKB1 | TXN | 1mdj_BA | 1mdj_A | 9190 | 6867 |
| TP53 | TP53BP1 | 1gzh_CD | 1gzh_D | 9187 | 6603 |
| CREBBP | IRF3 | 1zoq_CA | 1zoq_A | 9172 | 6809 |
| EGFR | ERRFI1 | 2rfd_AC | 2rfd_A | 9156 | 6799 |
| BCL2L1 | BECN1 | 2p1l_GH | 2p1l_G | 9154 | 6837 |
| RAF1 | RAP1A | 1gua_BA | 1gua_A | 9142 | 6622 |
| ARHGAP1 | CDC42 | 1am4_AD | 1am4_D | 9127 | 6716 |
| ARHGDIA | RAC2 | 1hh4_DA | 1hh4_D | 9121 | 6756 |
| ARHGDIA | CDC42 | 1doa_BA | 1doa_B | 9118 | 6716 |
| ARHGAP1 | CDC42 | 1grn_BA | 1grn_A | 9071 | 6716 |
| CALM2 | MARCKS | 1iwq_AB | 1iwq_A | 9068 | 6630 |
| ARHGAP1 | CDC42 | 1am4_BE | 1am4_E | 9062 | 6716 |
| MAX | MYC | 1nkp_BA | 1nkp_A | 9061 | 6577 |
| ARHGDIA | RAC2 | 1hh4_EB | 1hh4_E | 9030 | 6756 |
| TP53 | TP53BP1 | 1kzy_BD | 1kzy_D | 9010 | 6603 |
| HRAS | RASA1 | 1wq1_RG | 1wq1_R | 9002 | 6574 |

Instances are listed that have the druggability scores of >9,000 by the SVM model using all attributes and >6,500 by the SVM model using the top 10 attributes by F-score.

**Table S4: Small chemicals showing similarities to the hot spots of the SMAD4/SKI complex.**

| ZINC code | 2D structure | ZINC code | 2D structure |
|---|---|---|---|
| 2307 | 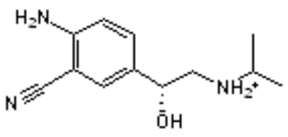 | 2539810 | 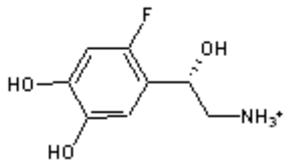 |
| 133303 | 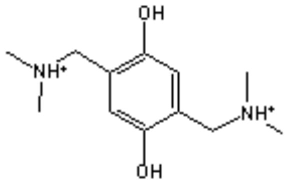 | 3830274 | 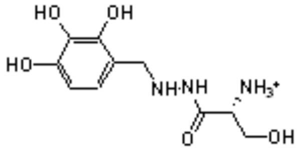 |
| 402980 | 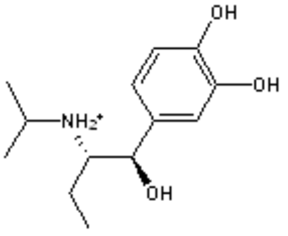 | 6525260 | 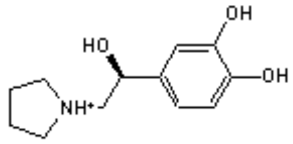 |
| 520267 | 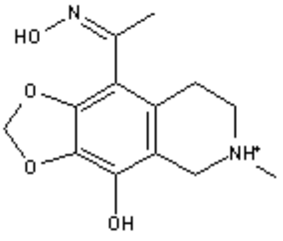 | 12411185 | 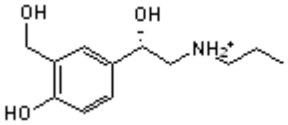 |
| 1760444 | 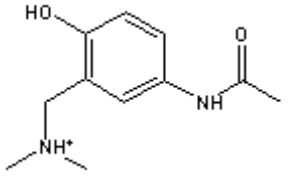 | | |
