# Supplementary material for: Assessing the druggability of protein-protein interactions by a supervised machine-learning method
Source: BMC Bioinformatics. 2009 Aug 25;10:263. doi: 10.1186/1471-2105-10-263 (PMC2739204; doi:10.1186/1471-2105-10-263)
Supplement: Additional file 4 — Supplementary methods. Definition and calculation methods of the PPI attributes. [file 1471-2105-10-263-S4.pdf]

Supplementary Methods

Definitions and calculation methods for the PPI attributes

Structural information

From the physicochemical properties of potential ligand-binding pockets located on protein-protein interfaces, 28 attributes were selected as ‘structural information’. The detection of the ligand-binding pockets is described in the Methods section of the text. Information on amino acids constituting the protein-protein interface was retrieved from the PDBsum database [1]. For the pockets considered, we calculated the following physicochemical properties.

Volume of the pockets was calculated by the software package Molecular Operating Environment [2] and used as one of the PPI attributes. Volume is one of the essential properties of a pocket when a researcher assesses whether or not a small ligand can bind to the pocket. If a pocket volume is too small, a small ligand could not bind to the pocket. If a pocket volume is too large, it is highly probable that various types of small ligands promiscuously bind to the pocket.

Two attributes related to accessible surface area (ASA) of the pockets were adopted. Pocket ASA is also an important property for assessing whether the pocket is more suitable as a drug target. Like the volume, adequate size of ASA of a pocket seems to be needed for small ligands to bind to the pocket with high affinity. The two attributes adopted are ASA of the pocket surface and the percentage of the pocket ASA to the ASA of the total surface of the polypeptide chain on which the pocket was detected. The ASA of total surface was calculated as the sum of ASA of all solvent-exposed amino acids of the polypeptide chain. The ASA of each amino acid of the polypeptide chain was calculated with the DSSP program [3]. If the calculated ASA of an amino acid is larger than 15% of the absolute value of ASA of the amino acid, we considered the amino acid as solvent-exposed [4].

To assess how compact a configuration of pocket-constituent atoms is in tertiary space, we adopted the compactness defined in [5] and used as an attribute. The compactness of a pocket is defined as the pocket volume divided by the pocket ASA [5].

In general, protein-protein interfaces have been thought to be nearly flat. In most of the

already-known PPIs targeted by small ligands, however, the interfaces seem to be concave rather than flat. To assess how flat a pocket is, we introduced our original measure, planarity, in the present study (Figure S1). The planarity is calculated based on tertiary coordinate data of pocket-constituent atoms and is defined as

$$planarity = 1 - \frac{d_1 + d_2}{\max(d_{ij})}, \qquad \text{(S1)}$$

where d_1 is the maximum of distances between least-squares plane (LSP) of pocket atoms and an atom 'above' the LSP, d_2 is the maximum of distances between the LSP and an atom 'below' the LSP, and d_{ij} is the distance between two pocket-constituent atoms, i and j. The planarity ranges from 0 (concave) to 1 (fully flat). As shown in Figure S1, the planarity is based on the ratio of d_1+d_2 to max(d_{ij}). Thus, large and small pockets having a shape similar to each other can yield almost the same value of the planarity. In this case, actual distances between pocket-constituent atoms as well as the distance ratio frequently become important factors. Therefore, the distance d_1+d_2 was also selected as an attribute.

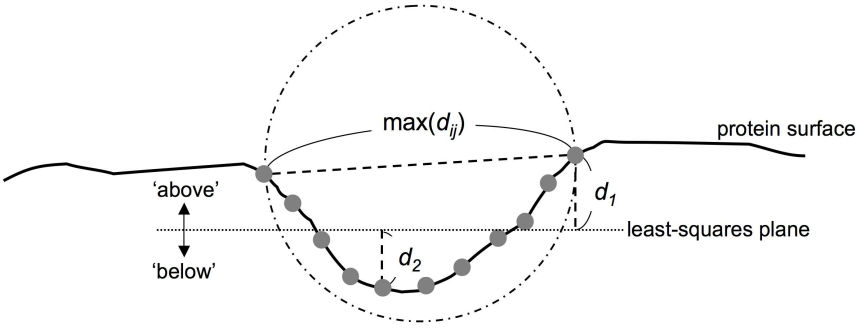

Figure S1. Definition of pocket planarity. A pocket is viewed horizontal to the least-squares plane (LSP) (shown by dotted line) of the pocket-constituent atoms. The planarity is calculated based on tertiary coordinate data of the pocket-constituent atoms (shown by filled gray circles). The maximum distance, max(d_{ij}), among all combinations of the pocket atoms is calculated. After the least-squares plane of the atoms is calculated, the distances, d_1 and d_2 (shown by broken lines), are calculated.

In some of the already-known target PPIs such as BCL2/BAK, ESR1/NCOA2, MDM2/TP53, and THRB/NCOA2, the ligand-binding pockets are those in which an α-helix from one protein interacts in the natural protein/protein complex. These pockets

seem very narrow when viewed from above the pocket surface. To assess how narrow a pocket is, we introduced a novel measure, narrowness, and used it as an attribute (Figure S2). First, all pocket atoms are projected to the LSP of the atoms and the projected coordinate data are used for the calculation. The narrowness is defined as

$$narrowness = 1 - \frac{d_4 + d_5}{d_3}, \qquad \text{(S2)}$$

where d_3 is the maximum of distances between atoms projected to the LSP of the pocket atoms, and d_4 and d_5 are defined as follows. Suppose that l_{ij} is a line passing through two atoms, i and j, giving the distance d_3. The d_4 is the maximum of distances between the l_{ij} and a projected atom 'right' of the l_{ij}. The d_5 is the maximum of distances between the l_{ij} and a projected atom 'left' of the l_{ij}. The narrowness ranges from 0 (full circle) to 1 (line). The distance d_4+d_5 was also used as an attribute.

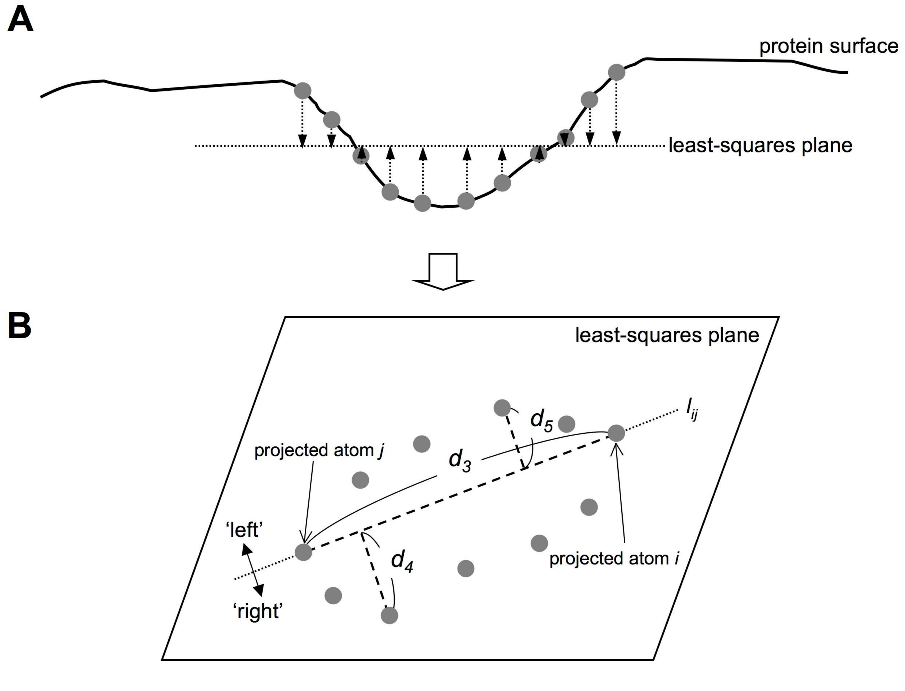

Figure S2. Definition of pocket narrowness. (A) All pocket atoms (shown by filled gray circles) are projected to the LSP. The narrowness is calculated based on the coordinate data of these projected atoms. (B) A bird's-eye view of the LSP to which the pocket atoms are projected. Maximum distance, d_3 (shown by broken lines), among all combinations of the projected atoms is calculated. In this case, the distance between two projected atoms i and j is the largest. Then, the distances, d_4 and d_5 (shown by broken lines), are calculated.

Previous studies [6,7] have shown that some amino acids are more preferred as 'hot spots' at protein-protein interfaces. We adopted amino acid composition on the pocket surface as attributes. To avoid any influence of amino acid composition bias in the full polypeptide chain, we defined the attributes as ratios of amino acid frequencies on the pocket surface to those on the total surface of the polypeptide chain. Only solvent-exposed amino acids were considered for the calculation.

Drug and chemical information

In several of the already-known target PPIs, one interacting partner is a protein that has been already targeted by a drug approved by the Food and Drug Administration (FDA). Several other already-known target PPIs are novel drug targets, and both interacting partners have no FDA-approved drug targeting them. To assess whether or not the fact that an interacting protein is an already-known drug target influences the selection of PPIs as drug targets, we utilized drug information on single proteins.

Sixteen attributes were selected as 'drug and chemical information'. The information on drugs and chemicals was retrieved from the DrugBank database [8]. In the DrugBank, each drug or chemical is categorized according to 8 criteria ('small molecule', 'biotech', 'approved', 'experimental', 'investigational', 'nutraceutical', 'withdrawn', and 'illicit'). For example, aspirin is categorized as 'small molecule' and 'approved', and interferon α-n3 as 'biotech', 'approved', and 'investigational'. For a PPI, we counted the number of drugs and chemicals targeting each of the two interacting proteins for every drug types. For a given PPI, we used the numbers for both interacting proteins; the larger one of the two numbers is described by attributes labeled as '*L*' (designating '*L*arge') and the smaller one by attributes labeled as '*S*' (designating '*S*mall'). If the two numbers were the same, we used the same number for both '*L*' and '*S*' attributes.

Functional information

Twenty-five attributes were selected as 'functional information'.

One attribute was selected from the information on human diseases registered in the OMIM database [9]. Information on human diseases caused when a protein is genetically or somatically mutated is an essential datum to assess the druggability of the protein. This attribute is scored as 1 if both two interacting proteins of a PPI are implicated in OMIM-registered diseases (not limited to the same disease). The attribute

is scored as 0 if only one interacting protein is implicated in a disease or if neither interacting protein is implicated in diseases.

We selected two attributes from the information on the number of interacting proteins. To repress a disease state by drugs, it could be desirable in some cases to target proteins that function as 'hubs' in PPI networks or biological pathways. In other cases, targeting proteins that function in a peripheral part of network or pathway could be more feasible for the treatment of a disease. After the PPI network was constructed based on the human PPI data in the Entrez Gene database [10], the numbers of all interacting proteins of each partner of a PPI were counted. As for the attributes in drug and chemical information, the numbers of interacting proteins for the two partners were designated as '*L*' and '*S*'. If the two numbers were the same, we used the same number for both '*L*' and '*S*' attributes.

Three attributes were selected from the information on biological pathways registered in the KEGG PATHWAY database [11]. As with the number of interacting proteins, proteins that function in a large number of biological pathways may be more desirable targets for therapeutic intervention for some diseases, while proteins involved in a limited number of pathways may be more desirable targets for other diseases. For a given PPI, we counted the number of pathways in which each interacting protein is involved; the larger of these two numbers was defined as '*L*' and the smaller as '*S*'. In addition, the number of pathways in which both partner proteins are involved was counted and used as an attribute.

To assess the degree of similarity in biological functions of the two interacting proteins in a PPI, we utilized Gene Ontology (GO) [12]. Based on the GO terms assigned to the interacting proteins, three attributes were calculated. We calculated the identity scores of GO terms between the two interacting proteins. The identity score ($S(i)_{GO}$) between a protein pair i is defined as

$$S(i)_{GO} = \sum_j L_j \cdot n_{ij} \;, \qquad \text{(S3)}$$

where L_j is the jth level of GO hierarchy (in the present study, L_j = 1, 2, 3, ..., 20, from the top level term (L_j = 1) to a specific term (L_j > 1)) and n_{ij} is the number of shared identical GO terms in the jth level between a protein pair i [13]. The scores for the three

GO categories (cellular component, molecular function, and biological process) were calculated.

Four attributes were selected from the information on the number of paralogs. The number of paralogs is an important factor for a protein to be selected as drug target, when a researcher considers adverse effects of a drug caused by binding of the drug to non-target paralogs. Target proteins with large numbers of paralogs may be associated with more severe adverse effects. The information on paralogs was retrieved from the KEGG ORTHOLOGY database [11] and the PIRSF database [14]. For each database, the numbers of paralogs of the two interacting proteins were counted. The numbers were used as '*L*' and '*S*'.

Twelve attributes were selected from the information on gene expression profiles in the UniGene database [15]. For a protein to be selected as drug target, it is more advantageous if the protein functions in the limited number of tissues/organs including disease-developing ones. Because of a scarcity of information on protein expression profiles in humans, we utilized gene expression profiles. In UniGene, a gene has three expression profiles based on 'health state', 'body sites', and 'developmental stage'. For genes encoding the proteins in the PPIs, we counted the numbers of health states, body sites, and developmental stages in which each gene is expressed. Because PPIs have the two numbers, these were used as '*L*' and '*S*'. In addition, the numbers of health states, body sites, and developmental stages in which both genes are expressed were also counted and used as attributes.

Furthermore, to assess the degree of similarity between the gene expression profiles of the two interacting proteins in a PPI, we calculated similarity scores of expression profiles between the two genes. For this purpose, the expression profiles were converted to binary mode (expressed = 1; not expressed = 0). The similarity score ($S(i)_{expression}$) between a gene pair i (composed of genes a and b) is based on the Dice's coefficient [16] and defined as

$$S(i)_{expression} = \frac{2n_{a=1,b=1}}{n_{a=1,b=0} + n_{a=0,b=1} + 2n_{a=1,b=1}}, \qquad \text{(S4)}$$

where $n_{a=1,b=1}$ is the number of health states, body sites, or developmental stages in which both genes (a and b) are expressed (a=1, b=1), $n_{a=1,b=0}$ is that in which one gene

expressed (a=1) and another not expressed (b=0), and $n_{a=0,b=1}$ is that in which one gene not (a=0) and another expressed (b=1). The score ranges from 0 (dissimilar) to 1 (similar). The scores were calculated for three expression profiles (health state, body sites, and developmental stage).

Supplementary references

1. Laskowski RA, Chistyakov VV, Thornton JM: **PDBsum more: new summaries and analyses of the known 3D structures of proteins and nucleic acids.** *Nucleic Acids Res* 2005, **33**:D266-268.
2. **Chemical Computing Group**
[http://www.chemcomp.com/]
3. Kabsch W, Sander C: **Dictionary of protein secondary structure: pattern recognition of hydrogen-bonded and geometrical features.** *Biopolymers* 1983, **22**:2577-2637.
4. Rost B, Sander C: **Conservation and prediction of solvent accessibility in protein families.** *Proteins* 1994, **20**:216-226.
5. Hajduk PJ, Huth JR, Fesik SW: **Druggability indices for protein targets derived from NMR-based screening data.** *J Med Chem* 2005, **48**:2518-2525.
6. Bogan AA, Thorn KS: **Anatomy of hot spots in protein interfaces.** *J Mol Biol* 1998, **280**:1-9.
7. Brinda KV, Kannan N, Vishveshwara S: **Analysis of homodimeric protein interfaces by graph-spectral methods.** *Protein Eng* 2002, **15**:265-277.
8. Wishart DS, Knox C, Guo AC, Cheng D, Shrivastava S, Tzur D, Gautam B, Hassanali M: **DrugBank: a knowledgebase for drugs, drug actions and drug targets.** *Nucleic Acids Res* 2008, **36**:D901-D906.
9. Hamosh A, Scott AF, Amberger JS, Bocchini CA, McKusick VA: **Online Mendelian Inheritance in Man (OMIM), a knowledgebase of human genes and genetic disorders.** *Nucleic Acids Res* 2005, **33**:D514-D517.
10. Maglott D, Ostell J, Pruitt KD, Tatusova T: **Entrez Gene: gene-centered information at NCBI.** *Nucleic Acids Res* 2007, **35**:D26-D31.
11. Kanehisa M, Araki M, Goto S, Hattori M, Hirakawa M, Itoh M, Katayama T, Kawashima S, Okuda S, Tokimatsu T, Yamanishi Y: **KEGG for linking genomes to life and the environment.** *Nucleic Acids Res* 2008, **36**:D480-D484.
12. The Gene Ontology Consortium: **The Gene Ontology project in 2008.** *Nucleic Acids Res* 2008, **36**:D440-D444.
13. Sugaya N, Ikeda K, Tashiro T, Takeda S, Otomo J, Ishida Y, Shiratori A, Toyoda A, Noguchi H, Takeda T, Kuhara S, Sakaki Y, Iwayanagi T: **An integrative *in silico* approach for discovering candidates for drug-targetable protein-protein interactions in interactome data.** *BMC Pharmacol* 2007, **7**:10.
14. Wu CH, Nikolskaya A, Huang H, Yeh LS, Natale DA, Vinayaka CR, Hu ZZ,

Mazumder R, Kumar S, Kourtesis P, Ledley RS, Suzek BE, Arminski L, Chen Y, Zhang J, Cardenas JL, Chung S, Castro-Alvear J, Dinkov G, Barker WC: **PIRSF: family classification system at the Protein Information Resource.** *Nucleic Acids Res* 2004, **32**:D112-D114.

15. Pontius JU, Wagner L, Schuler GD: **UniGene: a unified view of the transcriptome.** In *The NCBI Handbook.* Bethesda(MD): National Center for Biotechnology Information; 2003.
16. van Rijsbergen CJ: 1979. *Information retrieval.* London: Butterworths; 1979.
17. Renault L, Guilbert B, Cherfils J: **Structural snapshots of the mechanism and inhibition of a guanine nucleotide exchange factor.** *Nature* 2003, **426**:525-530.
18. Viaud J, Zeghouf M, Barelli H, Zeeh JC, Padilla A, Guibert B, Chardin P, Royer CA, Cherfils J, Chavanieu A: **Structure-based discovery of an inhibitor of Arf activation by Sec7 domains through targeting of protein–protein complexes.** *Proc Natl Acad Sci USA* 2007, **104**:10370-10375.
19. Oltersdorf T, Elmore SW, Shoemaker AR, Armstrong RC, Augeri DJ, Belli BA, Bruncko M, Deckwerth TL, Dinges J, Hajduk PJ, Joseph MK, Kitada S, Korsmeyer SJ, Kunzer AR, Letai A, Li C, Mitten MJ, Nettesheim DG, Ng S, Nimmer PM, O'Connor JM, Oleksijew A, Petros AM, Reed JC, Shen W, Tahir SK, Thompson CB, Tomaselli KJ, Wang B, Wendt MD, Zhang H, Fesik SW, Rosenberg SH: **An inhibitor of Bcl-2 family proteins induces regression of solid tumours.** *Nature* 2005, **435**:677-681.
20. Bruncko M, Oost TK, Belli BA, Ding H, Joseph MK, Kunzer A, Martineau D, McClellan WJ, Mitten M, Ng SC, Nimmer PM, Oltersdorf T, Park CM, Petros AM, Shoemaker AR, Song X, Wang X, Wendt MD, Zhang H, Fesik SW, Rosenberg SH, Elmore SW: **Studies leading to potent, dual inhibitors of Bcl-2 and Bcl-xL.** *J Med Chem* 2007, **50**:641-662.
21. Wu TYH, Wagner KW, Bursulaya B, Schultz PG., Deveraux QL: **Development and characterization of nonpeptidic small molecule inhibitors of the XIAP/caspase-3 interaction.** *Chem Biol* 2003, **10**:759-767.
22. Oost TK, Sun C, Armstrong RC, Al-Assaad AS, Betz SF, Deckwerth TL, Ding H, Elmore SW, Meadows RP, Olejniczak ET, Oleksijew A, Oltersdorf T, Rosenberg SH, Shoemaker AR, Tomaselli KJ, Zou H, Fesik SW: **Discovery of potent antagonists of the antiapoptotic protein XIAP for the treatment of cancer.** *J Med Chem* 2004, **47**:4417-4426.
23. Sun H, Nikolovska-Coleska Z, Yang CY, Xu L, Tomita Y, Krajewski K, Roller PP, Wang S: **Structure-based design, synthesis, and evaluation of conformationally**

constrained mimetics of the second mitochondria-derived activator of caspase that target the X-linked inhibitor of apoptosis protein/caspase-9 interaction site. *J Med Chem* 2004, **47**:4147-4150.

24. Sun H, Nikolovska-Coleska Z, Chen J, Yang CY, Tomita Y, Pan H, Yoshioka Y, Krajewski K, Roller PP, Wang S: **Structure-based design, synthesis and biochemical testing of novel and potent Smac peptido-mimetics.** *Bioorg Med Chem Lett* 2005, **15**:793-797.
25. Wendt MD, Sun C, Kunzer A, Sauer D, Sarris K, Hoff E, Yu L, Nettesheim DG, Chen J, Jin S, Comess KM, Fan Y, Anderson SN, Isaac B, Olejniczak ET, Hajduk PJ, Rosenberg SH, Elmore SW: **Discovery of a novel small molecule binding site of human survivin.** *Bioorg Med Chem Lett* 2007, **17**:3122-3129.
26. Osawa M, Swindells MB, Tanikawa J, Tanaka T, Mase T, Furuya T, Ikura M: **Solution structure of calmodulin-W-7 complex: the basis of diversity in molecular recognition.** *J Mol Biol* 1998, **276**:165-176.
27. Yokokura H, Osawa M, Inoue T, Umezawa I, Naito Y, Ikura M, Hidaka H: **Symmetric covalent linkage of N-(6-aminohexyl)-5-chloro-1-naphthalenesulfonamide (W-7) results in novel derivatives with increased inhibitory activities against calcium/calmodulin complex.** *Drug Des Discov* 1999, **16**:203-216.
28. Orner BP, Ernst JT, Hamilton AD: **Toward proteomimetics: terphenyl derivatives as structural and functional mimics of extended regions of an α-helix.** *J Am Chem Soc* 2001, **123**:5382-5383.
29. Harmat V, Böcskei Z, Náray-Szabó G, Bata I, Csutor AS, Hermecz I, Arányi P, Szabó B, Liliom K, Vértessy BG, Ovádi J: **A new potent calmodulin antagonist with arylalkylamine structure: crystallographic, spectroscopic and functional studies.** *J Mol Biol* 2000, **297**:747-755.
30. Li S, Gao J, Satoh T, Friedman TM, Edling AE, Koch U, Choksi S, Han X, Korngold R, Huang Z: **A computer screening approach to immunoglobulin superfamily structures and interactions: Discovery of small non-peptidic CD4 inhibitors as novel immunotherapeutics.** *Proc Natl Acad Sci USA* 1997, **94**:73-78.
31. Rodriguez AL, Tamrazi A, Collins ML, Katzenellenbogen JA: **Design, synthesis, and in vitro biological evaluation of small molecule inhibitors of estrogen receptor α coactivator binding.** *J Med Chem* 2004, **47**:600-611.
32. Van Duyne GD, Standaert RF, Karplus PA, Schreiber SL, Clardy J: **Atomic structure of FKBP-FK506, an immunophilin-immunosuppressant complex.** *Science* 1991, **252**:839-842.

33. Wilson KP, Yamashita MM, Sintchak MD, Rotstein SH, Murcko MA, Boger J, Thomson JA, Fitzgibbon MJ, Black JR, Navia MA: **Comparative X-ray structures of the major binding protein for the immunosuppressant FK506 (tacrolimus) in unliganded form and in complex with FK506 and rapamycin.** *Acta Crystallogr D Biol Crystallogr* 1995, **51**:511-521.
34. Wang T, Li BY, Danielson PD, Shah PC, Rockwell S, Lechleider RJ, Martin J, Manganaro T, Donahoe PK: **The immunophilin FKBP12 functions as a common inhibitor of the TGFβ family type I receptors.** *Cell* 1996, **86**:435-444.
35. Huse M, Chen YG, Massagué J, Kuriyan J: **Crystal structure of the cytoplasmic domain of the type I TGFβ receptor in complex with FKBP12.** *Cell* 1999, **96**:425-436.
36. Furet P, García-Echeverría C, Gay B, Schoepfer J, Zeller M, Rahuel J: **Structure-based design, synthesis, and X-ray crystallography of a high-affinity antagonist of the Grb2-SH2 domain containing an asparagine mimetic.** *J Med Chem* 1999, **42**:2358-2363.
37. Fretz H, Furet P, Garcia-Echeverria C, Schoepfer J, Rahuel J: **Structure-based design of compounds inhibiting Grb-SH2 mediated protein-protein interactions in signal transduction pathways.** *Curr Pharm Des* 2000, **6**:1777-1796.
38. Atabey N, Gao Y, Yao ZJ, Breckenridge D, Soon L, Soriano JV, Burke TR Jr, Bottaro DP: **Potent blockade of hepatocyte growth factor-stimulated cell motility, matrix invasion and branching morphogenesis by antagonists of Grb2 Src homology 2 domain interactions.** *J Biol Chem* 2001, **276**:14308-14314.
39. Ji T, Lee M, Pruitt SC, Hangauer DG: **Privileged scaffolds for blocking protein-protein interactions: 1,4-disubstituted naphthalene antagonists of transcription factor complex HOX-PBX/DNA.** *Bioorg Med Chem Lett* 2004, **14**:3875-3879.
40. Sarabu R, Cooper JP, Cook CM, Gillespie P, Perrotta AV, Olson GL: **Design and synthesis of small molecule interleukin-1 receptor antagonists based on a benzene template.** *Drug Design Discov* 1998, **15**:191-198.
41. Arkin MR, Randal M, DeLano WL, Hyde J, Luong TN, Oslob JD, Raphael DR, Taylor L, Wang J, McDowell RS, Wells JA, Braisted AC: **Binding of small molecules to an adaptive protein–protein interface.** *Proc Natl Acad Sci USA* 2003, **100**:1603-1608.
42. Thanos CD, Randal M, Wells JA: **Potent small-molecule binding to a dynamic hot spot on IL-2.** *J Am Chem Soc* 2003, **125**:15280-15281.
43. Fujii N, Haresco JJ, Novak KA, Gage RM, Pedemonte N, Stokoe D, Kuntz ID, Guy

RK: **Rational design of a nonpeptide general chemical scaffold for reversible inhibition of PDZ domain interactions.** *Bioorg Med Chem Lett* 2007, **17**:549-552.

44. Fry DC, Emerson SD, Palme S, Vu BT, Liu CM, Podlaski F: **NMR structure of a complex between MDM2 and a small molecule inhibitor.** *J Biomol NMR* 2004, **30**:163-173.
45. Grasberger BL, Lu T, Schubert C, Parks DJ, Carver TE, Koblish HK, Cummings MD, LaFrance LV, Milkiewicz KL, Calvo RR, Maguire D, Lattanze J, Franks CF, Zhao S, Ramachandren K, Bylebyl GR, Zhang M, Manthey CL, Petrella EC, Pantoliano MW, Deckman IC, Spurlino JC, Maroney AC, Tomczuk BE, Molloy CJ, Bone RF: **Discovery and cocrystal structure of benzodiazepinedione HDM2 antagonists that activate p53 in cells.** *J Med Chem* 2005, **48**:909-912.
46. Eaton SR, Cody WL, Doherty AM, Holland DR, Panek RL, Lu GH, Dahring TK, Rose DR: **Design of peptidomimetics that inhibit the association of phosphatidylinositol 3-kinase with platelet-derived growth factor-β receptor and possess cellular activity.** *J Med Chem* 1998, **41**:4329-4342.
47. Gao Y, Dickerson JB, Guo F, Zheng J, Zheng Y: **Rational design and characterization of a Rac GTPase-specific small molecule inhibitor.** *Proc Natl Acad Sci USA* 2004, **101**:7618-7623.
48. Siddiquee K, Zhang S, Guida WC, Blaskovich MA, Greedy B, Lawrence HR, Yip ML, Jove R, McLaughlin MM, Lawrence NJ, Sebti SM, Turkson J: **Selective chemical probe inhibitor of Stat3, identified through structure-based virtual screening, induces antitumor activity.** *Proc Natl Acad Sci USA* 2007, **104**:7391-7396.
49. Trosset JY, Dalvit C, Knapp S, Fasolini M, Veronesi M, Mantegani S, Gianellini LM, Catana C, Sundström M, Stouten PF, Moll JK: **Inhibition of protein–protein interactions: the discovery of druglike β-catenin inhibitors by combining virtual and biophysical screening.** *Proteins* 2006, **64**:60-67.
50. Estébanez-Perpiñá E, Arnold LA, Jouravel N, Togashi M, Blethrow J, Mar E, Nguyen P, Phillips KJ, Baxter JD, Webb P, Guy RK, Fletterick RJ: **Structural insight into the mode of action of a direct inhibitor of coregulator binding to the thyroid hormone receptor.** *Mol Endocrinol* 2007, **21**:2919-2928.
51. He MM, Smith AS, Oslob JD, Flanagan WM, Braisted AC, Whitty A, Cancilla MT, Wang J, Lugovskoy AA, Yoburn JC, Fung AD, Farrington G, Eldredge JK, Day ES, Cruz LA, Cachero TG, Miller SK, Friedman JE, Choong IC, Cunningham BC: **Small-molecule inhibition of TNF-α.** *Science* 2005, **310**:1022-1025.
52. Vu CB, Corpuz EG, Merry TJ, Pradeepan SG, Bartlett C, Bohacek RS, Botfield MC,

Eyermann CJ, Lynch BA, MacNeil IA, Ram MK, van Schravendijk MR, Violette S, Sawyer TK: **Discovery of potent and selective SH2 inhibitors of the tyrosine kinase ZAP-70.** *J Med Chem* 1999, **42**:4088-4098.

53. Irwin JJ, Shoichet BK: **ZINC - a free database of commercially available compounds for virtual screening.** *J Chem Inf Model* 2005, **45**:177-182.
54. Lipinski, CA: **Drug-like properties and the causes of poor solubility and poor permeability.** *J Pharmacol Toxicol Methods* 2000, **44**:235-249.
