# Supplementary material for: Assessing the druggability of protein-protein interactions by a supervised machine-learning method
Source: BMC Bioinformatics. 2009 Aug 25;10:263. doi: 10.1186/1471-2105-10-263 (PMC2739204; doi:10.1186/1471-2105-10-263)
Supplement: Additional file 6 — Figure S3. Discovery of small ligands showing similarities to the hot spots of the SMAD4/SKI complex. [file 1471-2105-10-263-S6.pdf]

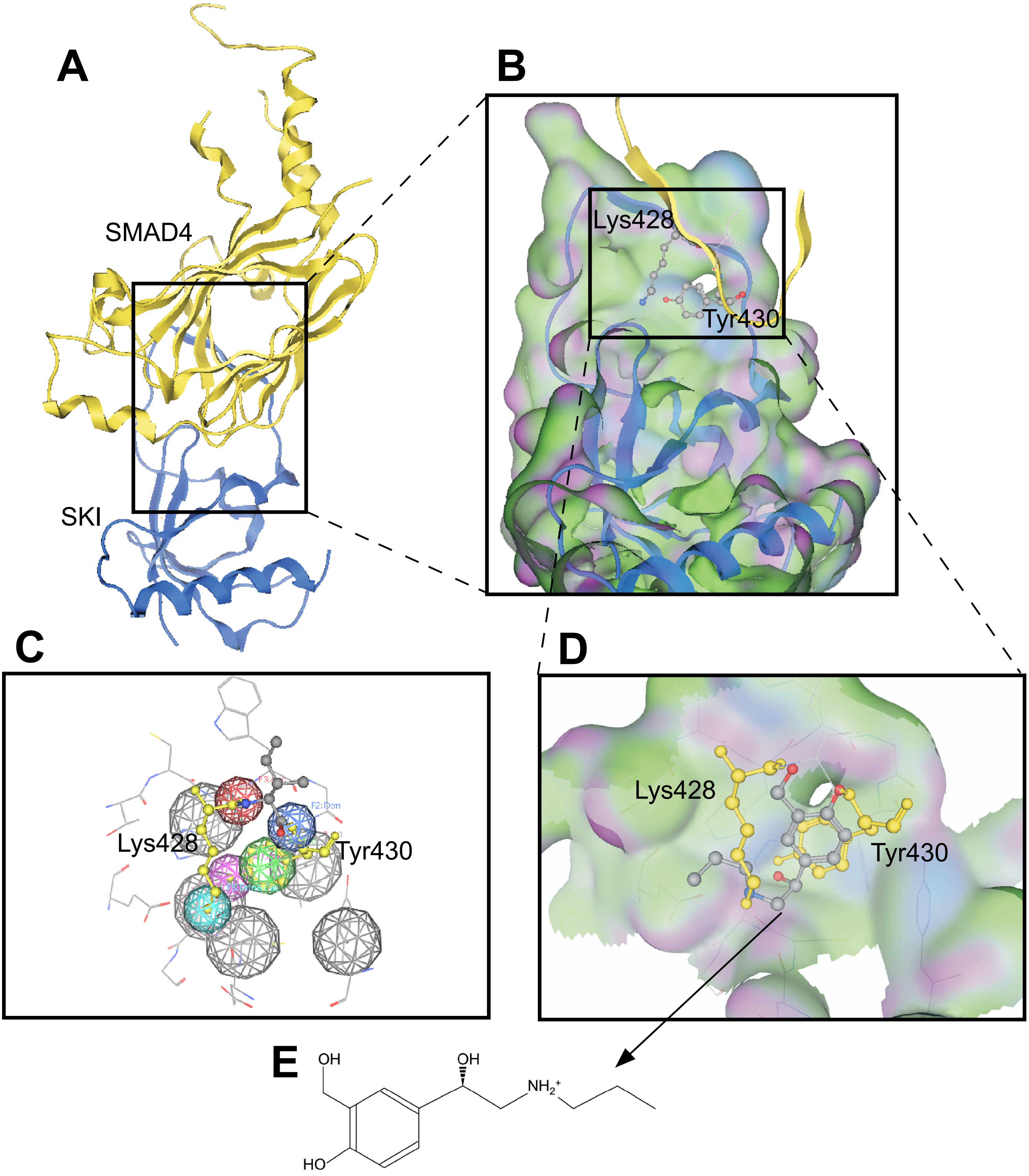

Figure S3. Discovery of small ligands showing similarities to the hot spots of the SMAD4/SKI complex. (A) Tertiary structure of the protein complex of the SMAD4/SKI [PDB:1MR1_BC]. (B) Hot spots (Lys428 and Tyr430 of the SMAD4) of the SMAD4/SKI used to create the pharmacophore and search for small ligands similar to them. (C) Pharmacophore query used to search for small ligands. Ten pharmacophoric points were set up. Each wireframe sphere corresponds to one of the query features set by us: green, aromatic rings; cyan, cation and hydrogen-bond donor; red, hydrogen-bond acceptor; blue, hydrogen-bond donor; purple, hydrogen-bond acceptor and donor; gray, excluded volume. Using this model, we searched for small ligands having a conformation satisfying all of the query features. Pharmacophore search was done against a subset of drug-like chemicals in the ZINC database [53]; this subset was composed of small chemicals satisfying 'Lipinski's rule of 5' [54] and included 146,195 chemicals after the selection of chemicals that differ from each other by the threshold of Tanimoto coefficient <90%. (D) Superimposition of one of the small ligands [ZINC:12411185] detected on the hot spots. (E) ZINC12411185. For references, see Additional file 5: Supplementary references.
